# UM171 expands distinct types of myeloid and NK progenitors from human pluripotent stem cells

**DOI:** 10.1038/s41598-019-43054-4

**Published:** 2019-04-29

**Authors:** Walatta-Tseyon Mesquitta, Matthew Wandsnider, HyunJun Kang, James Thomson, Oleg Moskvin, Kran Suknuntha, Igor I. Slukvin

**Affiliations:** 1 Wisconsin National Primate Research Center, University of Wisconsin, Madison, WI 53715 USA; 2 Morgridge Institute for Research, 330N. Orchard Street, Madison, WI 53715 USA; 3 Department of Molecular, Cellular, and Developmental Biology, University of California, Santa Barbara, CA 93106 USA; 4 Department of Cell and Regenerative Biology, School of Medicine and Public Health, University of Wisconsin, Madison, WI 53707 USA; 5 Department of Pathology and Laboratory Medicine, University of Wisconsin, Madison, WI 53792 USA; 6 Department of Pharmacology, Faculty of Science, Mahidol University, Bangkok, 10400 Thailand

**Keywords:** Cell growth, Lymphopoiesis

## Abstract

Scaling up blood cell production from hPSCs is critical to advancing hPSC technologies for blood transfusion, immunotherapy, and transplantation. Here we explored the potential of the HSC agonist pyrimido-indole derivative UM171, to expand hematopoietic progenitors (HPs) derived from hPSCs in chemically defined conditions. We revealed that culture of hPSC-HPs in HSC expansion conditions (SFEM with added TPO, SCF, FLT3L, IL3 and IL6) in the presence of UM171 predominantly expanded HPs with a unique CD34^+^CD41a^lo^CD45^+^ phenotype that were enriched in granulocytic progenitors (G-CFCs). In contrast, in lymphoid cultures on OP9-DLL4, in the presence of SCF, FLT3L, and IL7, UM171 selectively expanded CD34^+^CD45^+^CD7^+^ lymphoid progenitors with NK cell potential, and increased NK cell output up to 10-fold. These studies should improve our understanding of the effect of UM171 on *de novo* generated HPs, and facilitate development of protocols for robust granulocyte and lymphoid cell production from hPSCs, for adoptive immunotherapies.

## Introduction

Human pluripotent stem cells (hPSCs) have created alternative platforms for producing blood cells for transfusion, immunotherapies, and transplantation^1–4^. Advancing blood cell manufacturing from hPSCs and translating hPSC-based technologies to the clinic requires improving the scalability of blood cell production through enhancing hematopoietic differentiation of hPSCs and expanding lineage-committed hematopoietic progenitors (HPs). The pyrimido-indole derivative UM171 has been described as one of the most potent small molecules that stimulates HSC expansion *in vitro*^5^. UM171 selectively expands EPCR^+^ cord blood HSCs with sustained short- and long-term repopulation potential^6^, and mobilized peripheral blood HSCs following lentiviral transduction^7^. In addition, UM171 increases production of CD34^+^CD43^+^ HPs when added to hPSC differentiation cultures^8^. However, the effect and mechanism of UM171 action on hPSC-derived HPs has not been explored. CD34^+^CD43^+^ HPs generated from hPSCs are composed of a mixture of different types of progenitors, including lin^−^CD34^+^CD45^−^ and lin^−^CD34^+^CD45^+^ multipotential progenitors, and CD235a^+^CD41a^+^CD45^−^, and CD235a^+^CD41a^+^CD45^+^ progenitors, with erythro-megakaryocytic potential^9–11^. It remains unclear whether UM171 uniformly expands the most primitive lin^−^CD34^+^CD43^+^ multipotential progenitors, or selectively affects progenitors of a particular cell lineage, and whether it affects proliferation or programmed cell death of hPSC-derived HPs.

In this article, we evaluated the effect of UM171 on expansion and hematopoietic differentiation of HPs that were generated from hPSCs in chemically defined serum- and feeder-free conditions^10^. We revealed that culture of HPs in HSC expansion medium in the presence of UM171 predominantly expands CD34^+^CD41a^lo^CD45^+^ HPs enriched in G-CFCs. In contrast, in lymphoid cultures on OP9-DLL4, UM171 preferentially expands CD34^+^CD45^+^CD7^+^ lymphoid progenitors with robust NK potential. UM171-mediated expansion of HPs was associated with increased proliferation and decreased apoptosis.

These studies should improve our understanding of the effect of UM171 on *in vitro* generated HPs and facilitate development of protocols for robust granulocyte and lymphoid cell production from hPSCs for adoptive immunotherapies.

## Results

### UM171 preferentially expands hematopoietic progenitors with a unique CD34^+^CD41a^lo^CD45^+^ phenotype enriched in G-CFCs

**Figure 1:**
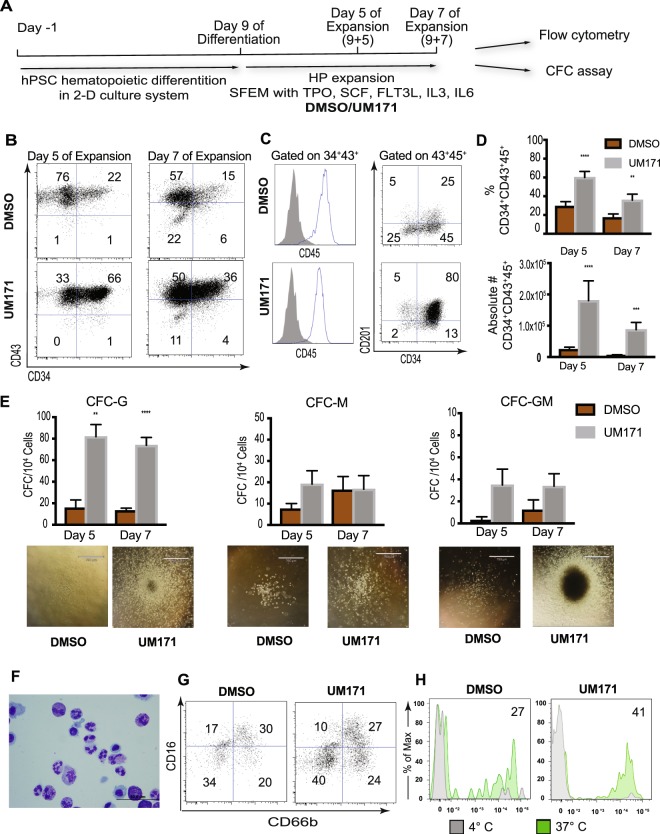
UM171 effect on expansion of CD34^+^CD43^+^ hPSC-derived HPs. (**A**) Schematic diagram of protocol used for expansion of HPs generated on day 9 H1 hESC differentiation in chemically defined conditions. (**B**) Representative dot plots show CD34 and CD43 expression following 5 and 7 days of expansion with UM171 or DMSO (control). (**C**) Histograms show that most of the cells in expansion cultures acquire CD45 expression. Dot plot demonstrates enhancing effect of UM171 on CD201 expression by CD34^+^ cells. (**D**) UM171 effect on % and absolute numbers of CD34^+^CD43^+^CD45^+^ HPs in cultures of hESC-derived CD43^+^ cells expanded for 5 and 7 days. Results are mean ± SEM for 7 independent experiments (Day 5), and 6 independent experiments (Day 7). **p < 0.01, ***p < 0.001 (**E**) CFC potential of expanded cells. Results are mean ± SEM for 7 independent experiments (Day 5), and 6 independent experiments (Day 7). **p < 0.01, ***p < 0.001. Representative images of colonies from HPs expanded with and without UM171 are shown. Image bar is 790 μM. (**F**) Cytospin showing morphology of granulocytes generated from UM171 expanded hematopoietic progenitors. Image bar is 50 μM. (**G**) Phenotype of neutrophils generated from hematopoietic progenitors expanded for 3 days with DMSO or UM171. (**H**) Phagocytosis of zymosan particles by neutrophils. Plots show histograms for cells incubated at 4 °C (filled gray; nonspecific binding control) and 37 °C (filled green). Percentages of FITC-positive cells at 37 °C minus nonspecific binding control at 4 °C are shown.

To understand the effect of UM171 on hPSC-derived HPs and provide mechanistic insight on its action, we performed hematopoietic differentiation of H1 hESCs in defined feeder- and serum-free conditions for 9 days to generate HPs^10^. We then cultured them in SFEM medium supplemented with cytokines that support expansion of HSCs (TPO, SCF, FLT3L, IL3 and IL6), and with UM171 or DMSO (negative control) (Fig. 1A). As shown in Fig. 1B–D, the percentages and absolute numbers of CD34^+^CD43^+^ HPs almost all of which also co-expressed CD45 were significantly higher in cultures with UM171, as compared to controls (DMSO). Overall, cultures with UM171 generated up to 10-fold higher numbers of CD34^+^CD43^+^CD45^+^ HPs, as compared to control cultures. Because previous studies had demonstrated that UM171 induces expression of endothelial protein C receptor (EPCR, also known as CD201) in cord blood HSC expansion cultures^6^, we analyzed the expression of this receptor in hPSC-derived HPs that were expanded in HSC conditions. As shown in Fig. 1C, expansion of hPSC-derived hematopoietic cells with UM171 was also associated with induction of CD201 expression in CD34^+^CD45^+^ HPs.

Assessment of the CFC potential of expanded cells revealed that UM171 had the most dramatic effect on G-CFCs (Fig. 1E). In addition, we noted that myeloid CFCs generated from UM171 expanded HPs were much larger and denser, thereby suggesting their higher potency (Fig. 1E). The effect of UM171 on the expansion of CD34^+^CD43^+^ HPs and G-CFCs was further confirmed using other H9 hESC and DF19-9-7T fibroblast-derived iPSC lines (Supplemental Fig. 1A–D). To confirm granulocytic potential of expanded cells, we cultured them with G-CSF to induce differentiation towards neutrophils. As shown in Fig. 1F–H, cells generated in this condition displayed typical neutrophil morphology and phenotype, and were capable of ingesting zymosan particles.

**Figure 2:**
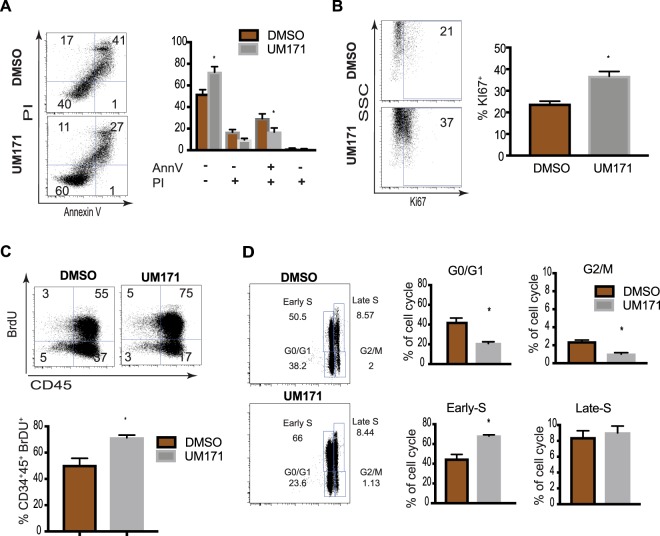
UM171 enhances survival and proliferation of CD34^+^CD43^+^ hPSC-derived HPs. (**A**) Apoptosis evaluation using annexin V staining in cultures of hPSC-derived HPs expanded for 5 days. Bars are mean ± SEM for 6 independent experiments. (**B**–**D**) Assessment of proliferative potential of CD34^+^ HPs expanded for 5 days with UM171 or DMSO using Ki67 staining (**B**), BrdU assay (**C**) and cell cycle analysis (**D**). Bars in (**B**–**D**) are mean ± SEM for 3 independent experiments. *p < 0.05. All dot plots show CD34^+^CD43^+^ gated cells.

Flow cytometric analysis of apoptosis by annexin V assay demonstrated an increased number of viable cells and a decreased number of apoptotic, especially late apoptotic cells (AnnexinV^+^PI^+^), in UM171 cultures, as compared to controls (Fig. 2A). In addition, UM171 expansion of HPs was associated with increased proliferation, as determined by BrdU assay and Ki67 staining (Fig. 2B,C). Extending these observations, cell cycle analysis revealed that UM171 predominantly increases the proportion of HPs in the early S phase of the cell cycle (Fig. 2D). Similar findings were obtained with CD34^+^CD43^+^ cells generated from H9 hESCs and DF19-97T hiPSCs, and that were expanded in HSC expansion conditions in the presence of UM171 (Supplemental Fig. 1E–J).

**Figure 3:**
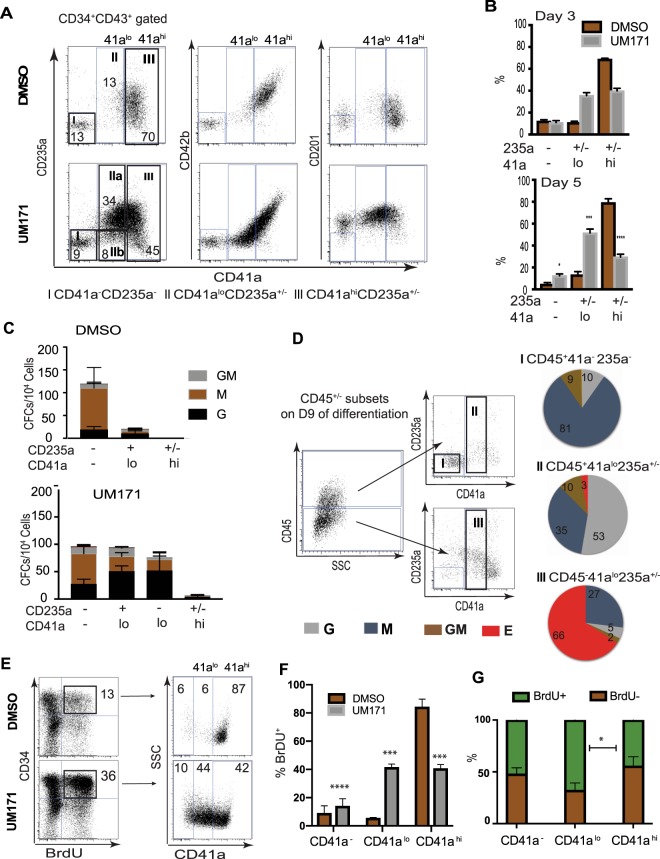
Flow cytometric and functional analysis of major cellular subsets amplified in HSC expansion conditions with UM171. (**A**) Representative dot plots show phenotype of major subsets of CD34^+^CD43^+^ cells identified in cultures after 3 days of expansion and gates used for their sorting (CD235a vs CD41a dot plots on the left). (**B**) Bar depicts mean  ±﻿ SEM percentage of each subset within the CD34^+^CD43^+^ population at day 3 (n = 4) and day 5 (n = 7) of expansion. (**C**) CFC composition for each sorted population. Results are mean ± SEM for 6 independent experiments. (**D**) Identification and characterization of CD41a^lo^ population within CD45^+^ hematopoietic progenitors formed on day 9 of differentiation, before initiation of expansion. Dot plots show gates used for analysis. Pie graphs show relative proportions of each type of CFC within sorted populations. (**E**) Representative dot plots show phenotype of BrdU^+^ proliferating cells, after gating CD45^+^ cells from DMSO and UM171 expanded HPs. (**F**) Percentages of CD41a^−^, CD41a^lo^, and CD41^hi^ subsets within BrdU^+^ proliferating cells after gating on the CD34^+^CD45^+^ population. Results are mean ± SEM (n = 3). **p < 0.01, ***p < 0.001. (**G**) Percentages of BrdU^+^ and BrdU^−^ cells within indicated subsets after 5 days of expansion; Results are mean ±  SEM (n = 3). *p < 0.05.

Our prior studies revealed that CD34^+^CD43^+^ HPs generated from hPSCs on day 9 of differentiation are composed of four major subsets: (1) CD235a^+^CD41a^+^CD45^−^ and (2) CD235a^+^CD41a^+^CD45^+^ progenitors with erythro-megakaryocytic potential; and (3) lin^−^CD45^−^ and (4) lin^−^CD45^+^ multipotential HPs^9–11^. To determine which types of progenitors are affected by UM171, we performed flow cytometric analysis of CD34^+^CD43^+^ cells from expansion cultures. As shown in Fig. 1C, these cells also coexpressed CD45, i.e. displayed a CD34^+^CD43^+^CD45^+^ phenotype. We found that in control conditions, CD34^+^ cells become more differentiated, acquiring a CD41a^hi^CD42b^+^ phenotype. In contrast, cultures with UM171 preferentially expanded a unique CD41a^lo^235a^+/−^CD42b^−^ cell population, which coexpressed CD201 (Fig. 3A,B). In addition, UM171 treated cultures retained HPs with CD41a^−^CD235a^−^CD34^+^ phenotype, while in control conditions this population mostly disappeared by day 5 of expansion (Fig. 3B). To determine the phenotype associated with amplified HPs, we performed sorting of three major subsets of CD34^+^CD43^+^CD45^+^ HPs from UM171 and DMSO cultures, as shown in Fig. 3A. Because the CD41a^lo^ population in UM171 conditions included a sizable proportion of CD235a^−^ cells, we additionally subdivided this population into CD235a^+^ and CD235a^−^ subsets (Fig. 3A). Assessment of the CFC potential of sorted subsets revealed that in UM171 cultures, most CFCs were associated with the CD41a^lo^235a^+/−^CD42b^−^ and CD41a^−^CD235a^−^ phenotypes within total CD34^+^CD43^+^CD45^+^ cells, while the CD41a^hi^CD42b^+^ population was mostly devoid of myeloid CFCs (Fig. 3C). Separation of CD41a^lo^ cells into CD235a^+^ and CD235a^−^ subsets did not reveal substantial differences in myeloid CFCs between these populations. Interestingly, we found that majority of myeloid CFCs within CD41a^lo^235a^+/−^CD42b^−^ population were G-CFCs. Thus, we concluded that in cultures with HSC expansion cytokines, UM171 promotes development of a progenitor population with a CD34^+^CD41a^lo^CD45^+^ phenotype enriched in unipotential granulocytic progenitors, G-CFCs.

Although CD41a is considered a megakaryocytic lineage marker, it is also expressed by the earliest embryonic HPs and HSCs in mice^12–16^. In hPSC differentiation cultures, the vast majority of CD41a^+^ cells coexpress CD235a, a marker associated with the erythroid lineage^11,17,18^. CD41a^+^CD235a^+^ cells collected from hPSC-OP9 differentiation cultures possess predominantly E- and Mk-CFC potentials^11,17^. However, the CD235a^+^CD41a^+^ population obtained in 2D serum- and feeder-free conditions contains myeloid CFCs^10,18^, which are mostly associated with the CD235a^+^CD41a^+^CD45^+^ fraction^10^. Thus, it is possible that UM171 selectively amplifies a CD34^+^CD41a^lo^CD235a^+/−^CD45^+^ G-CFC enriched population, which is already formed during differentiation in serum- and feeder-free defined conditions. Indeed, flow cytometric analysis on day 9 of differentiation confirmed the presence of a small cell population with CD41a^lo^CD235a^+/−^CD45^+^ phenotype (Fig. 3D). Sorting subsets based on CD41a and CD45 expression demonstrated G-CFC enrichment in the CD41a^lo^CD45^+^ fraction. In addition, cell cycle analysis revealed that almost 90% of proliferating cells in DMSO expansion cultures were CD41a^hi^, while UM171 cultures showed approximately equal proportions of CD41a^lo^ and CD41a^hi^ cells within the BrdU^+^ fraction, with up to 7-fold higher frequency of proliferating CD41a^lo^ HPs, as compared to DMSO control (Fig. 3E,F). Further analysis of frequency proliferating cells within each subset in UM171 conditions, demonstrated the highest frequency of proliferating cells within the CD34^+^CD41a^lo^CD45^+^ population (Fig. 3G). Altogether, these findings suggest that the CD41a^lo^CD235a^+/−^CD45^+^ population enriched in G-CFCs arises from hPSCs during differentiation in defined conditions, and that UM171 selectively amplifies and maintains this population, rather than induces its *de novo* formation.

**Figure 4:**
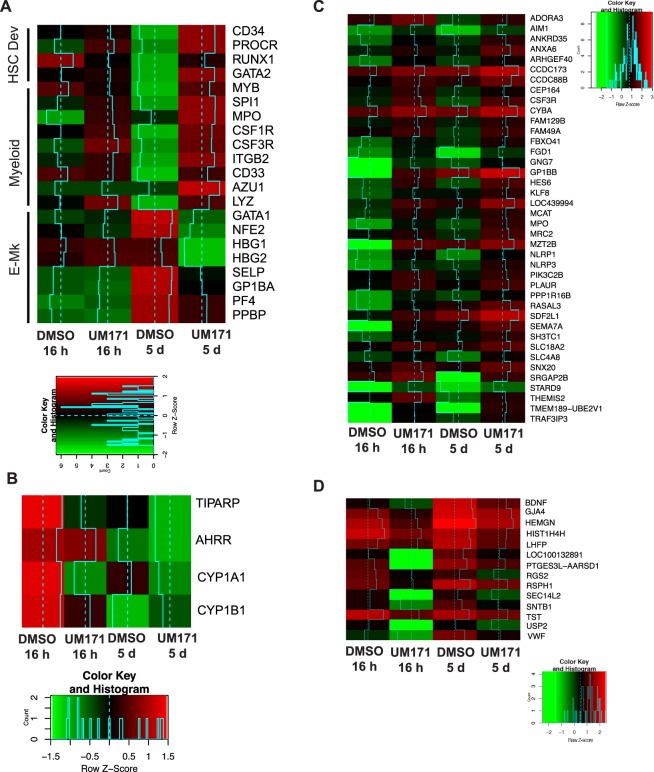
Changes in gene expression following expansion of CD34^+^CD235a^+^CD41a^+^ progenitors in HSC expansion conditions. (**A**) Heatmap shows changes in expression of genes associated with HSC, myeloid, and erythro-megakaryocytic lineage development. (**B**) Heatmap shows expression of AhR targets in corresponding expansion conditions. (**C**,**D**) Heatmaps showing commonly upregulated or downregulated genes that demonstrated more than 2-fold change in cultures expanded with UM171 for 16 hrs and 5 days.

To analyze changes in molecular profile induced by UM171, we isolated CD34^+^CD235^+^CD41a^+^ cells on day 9 of differentiation, cultured them in HSC expansion conditions with UM171 or DMSO for 16 hours and 5 days, and then performed RNAseq analysis of expanded cells. Consistent with our phenotypic and functional analysis, RNAseq analysis revealed upregulation of *CD34*, *PROCR*, *RUNX1,* and *GATA2* genes associated with HSC development, along with upregulation of myeloid-lineage associated genes and down-regulation of erythro-megakaryocytic genes (Fig. 4A). Similar to previously reported findings with cord blood HSCs^5^, UM171 did not affect the expression of aryl-hydrocarbon receptor target genes, which are involved in expansion of HSCs in the presence of another potent human HSC agonist, SR1^19^ (Fig. 4B).

Because UM171 preferentially amplifies progenitors with G-CFC potential, the major changes in RNAseq profile may reflect enrichment of lineage-committed cells, rather than downstream targets of UM171. Therefore, to better understand a direct effect of UM171 on HPs, we searched for genes that were upregulated by at least 2-fold after 16 hours, and remained upregulated by at least 2-fold after 5 days of expansion. This analysis revealed 40 upregulated and 14 downregulated genes that were common to both, early (16 h) and late (5 days) expansion groups (Fig. 4C). The group of upregulated common genes included genes involved in granulocytic lineage development (G-CSF receptor (*CSF3R*) and *MPO)*, and inflammatory response (*NLRP1*, *NLRP3*, *THEMIS2,* and *SEMA7*), suggesting that some effects of UM171 could be related to activation of inflammation associated pathways. The group of downregulated genes included genes highly expressed in megakaryocytes (*BDNF* and *VWF*) and the *HEMGN* gene, which increases transcriptional activity of GATA1 and promotes erythroid development^20^.

### UM171 enhances NK production from hPSC-derived HPs through expansion of CD34^+^CD7^+^ NK cell progenitors

**Figure 5:**
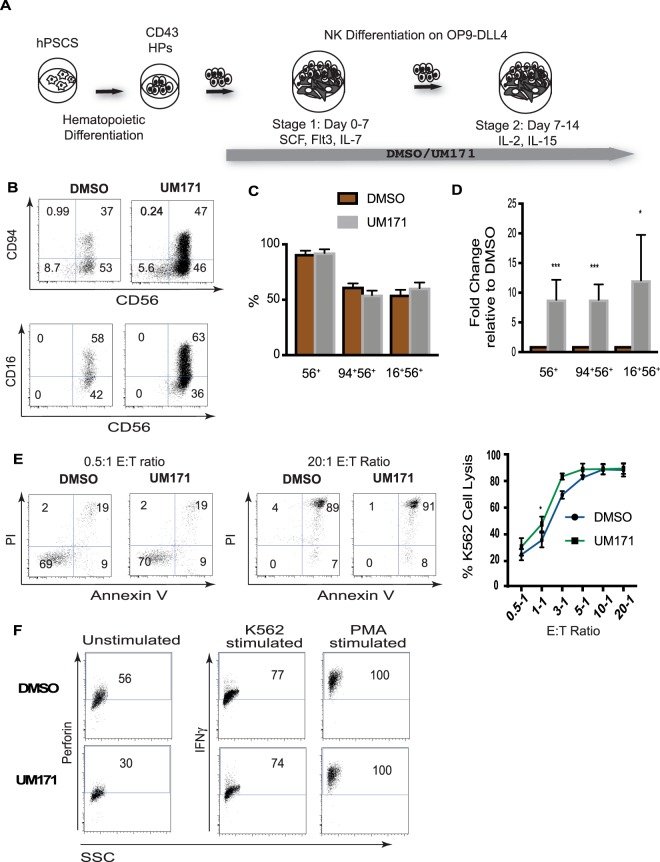
Effect of UM171 on NK cell production from hPSC-derived HPs. (**A**) Schematic diagram of protocol used for NK cell differentiation of hPSC-derived HPs. (**B**) Representative flow cytometry dot plots displaying NK cell differentiation. (**C**,**D**) Percentages and fold change in absolute numbers of NK cells. Bars are mean ± SEM for at least 6 independent experiments. **p < 0.01, ***p < 0.001. (**E**) Cytotoxicity assay against K562 targets. Representative dot plots and dose dependent cell lysis curves (mean ± SEM for n = 3) are shown. (**F**) Expression of perforin in unstimulated NK cells and INFγ following stimulation of NK cells with K562 or PMA.

To assess the effect of UM171 on NK cell differentiation, we collected HPs from hPSC cultures on day 9 of differentiation, and cultured them on OP9-DLL4, with or without UM171, as shown in Fig. 5A. After 14 days, these cultures generated CD56^+^ NK cells that expressed CD94 and CD16, markers of mature NK cells. Although UM171 had no effect on the percentages of NK cells or their phenotype, the absolute numbers of NK cells generated with UM171 were approximately 10-fold higher, as compared to DMSO controls (Fig. 5B–D). NK cells generated with UM171 possessed strong cytotoxicity, expressed perforin, and upregulated IFNγ production, following stimulation with K562 or PMA (Fig. 5E,F). These findings suggest that UM171 increases NK production through expansion, without affecting NK differentiation and function. Similar findings were obtained with HPs generated from H9 hESCs and the IISH2i-BM9 iPSC line that was derived from bone marrow mononuclear cells (Supplemental Fig. 2).

**Figure 6:**
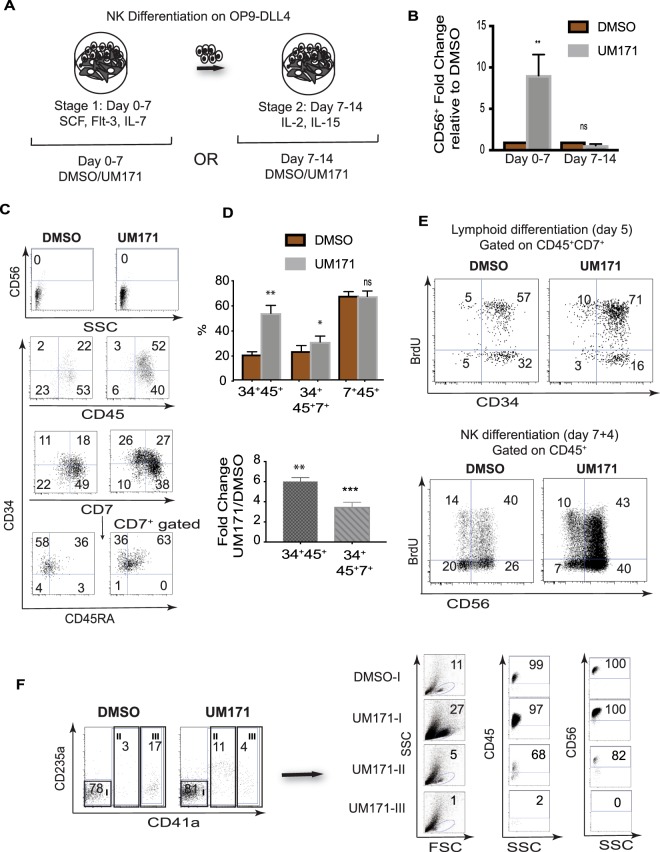
UM171 preferentially amplifies CD34^+^CD7^+^CD45^+^ NK cell progenitors. (**A**) Schematic diagram of experiments used to identify stage of NK development affected by UM171. (**B**) The effect of UM171 addition at stages 1 and 2 of NK cell differentiation. Bars are mean ± SEM for 3 independent experiments. (**C**) Phenotypic features of lymphoid progenitors generated in lymphoid cultures in the presence of UM171 or DMSO. (**D**) Upper panel shows the effect of UM171 on the proportion of cells with lymphoid progenitor phenotype on day 7 of NK differentiation (stage 1). Lower panel shows fold change in absolute numbers of CD34^+^ progenitors in cultures with UM171, as compared to DMSO controls. Bars are mean ± SEM for 3 independent experiments. **p < 0.01, ***p < 0.001. (**E**) Analysis of cell proliferation using BrdU assay in NK differentiation cultures, on days 5 and 11 of differentiation. (**E**) FAC-Sorting of cellular subsets from CD43^+^ HPs, on day 7 of lymphoid culture (stage 1), and assessment of their NK cell potential.

Our NK cell differentiation protocol is composed of two stages (Fig. 6A). At stage 1, HPs are cultured with SCF, FLT3L, and IL-7, to induce lymphoid commitment. At stage 2, cells are transferred to new OP9-DLL4, and cultured with IL-2 and IL-15, to induce NK differentiation. To determine the stage affected by UM171, we added UM171 during the first 7 or the final 7-14 days of NK cell differentiation cultures (Fig. 6A). As shown in Fig. 6B, UM171 exerted its effect only when added during the first 7 days, demonstrating that addition of UM171 during the initial lymphoid differentiation stage is sufficient to amplify terminal NK yields, and suggesting that UM171 may potentiate expansion of NK progenitors, rather than their differentiation into mature NK cells. Phenotypic analysis of cells generated during days 0–7 of NK cultures revealed that UM171 predominantly expands CD34^+^CD45^+^ HPs that are enriched in cells expressing CD7^+^ and CD45RA lymphoid progenitor markers (Fig. 6C,D). Cell cycle analysis with BrdU demonstrated that UM171 increases the proportion of proliferating CD34^+^CD45^+^CD7^+^ progenitors, while having no effect on NK maturation cultures (Fig. 6E), consistent with our observation of a predominant effect of UM171 on the lymphoid progenitor stage.

Most of the cells in lymphoid cultures, in contrast to cultures in HSC expansion conditions, were lacking CD41a and CD235a, although small proportions of cells with CD41a^lo^ and CD41a^hi^ phenotype were also detected (Fig. 6F). To determine whether lymphoid progenitor potential associated with CD41a^−^, CD41a^lo^, or CD41a^hi^ fractions, we sorted these cell populations, and assessed their NK cell potential. These studies revealed that the most robust NK potential resided in the CD41a^−^ fraction. No NK cell potential was detected in the CD41^hi^ fraction. Although the CD41a^lo^ fraction in UM171 conditions produced CD56^+^ NK cells, the efficiency of NK cell differentiation from this population was much lower (Fig. 6F). Thus, we concluded that in lymphoid conditions, UM171 expands a phenotypically and functionally different progenitor population with lymphoid potential.

## Discussion

**Figure 7:**
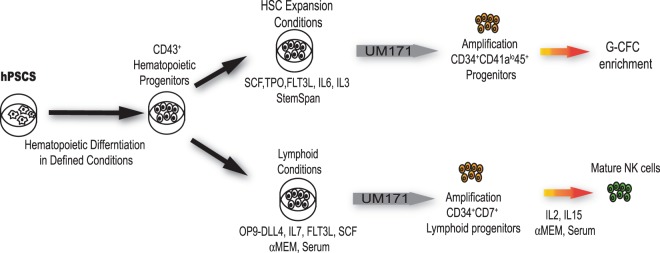
Schematic diagram summarizing the effect of UM171 on expansion of hPSC-derived HPs in HSC and lymphoid expansion conditions.

Scaling up blood cell production from hPSCs is critical for advancing hPSC technologies for blood transfusion, immunotherapies, and transplantation. This can be achieved through improving the efficiency of hematopoietic differentiation from hPSCs and subsequent amplification of HPs using cytokines, growth factors, and small molecules, or by overexpression of transcription factors that support self-renewal of the most primitive multipotential progenitors or lineage-restricted HPs. In the present study, we sought to explore the possibilities for enhancing blood production from hPSCs through the expansion of HPs generated from hPSCs in chemically defined conditions. Specifically, we focused on the pyrimido-indole derivative UM171, a small molecule that is known as one of the most potent enhancers of HSC expansion^5^. Here, we established that culture of hPSC-derived HPs in HSC expansion conditions with UM171 preferentially expands a CD34^+^CD41a^lo^CD45^+^ population that is enriched in G-CFCs. Although we observed an increased number of CD34^+^CD41a^−^CD235a^−^CD45^+^ multipotential HPs in these conditions, these cells comprised a relatively small fraction within the entire CD34^+^ population. In lymphoid cultures, UM171 preferentially expanded a CD34^+^CD45^+^CD7^+^ population with NK cell potential, while having a minimal effect on expansion of the CD34^+^CD41^lo^CD45^+^ population (Fig. 7).

The mechanisms of UM171 action remain unknown. It has been shown that UM171 upregulates expression of PROCR, which is involved in pathways regulating HSC retention in bone marrow^21^, and marks a highly enriched HSC population in expansion cultures^5^.

In contrast to another HSC expansion molecule SR1, which predominantly acts through inhibition of aryl hydrocarbon receptor (AHR)^19^, UM171 does not affect pathways associated with AHR^5^. Similar to findings with cord blood HSCs, we found that UM171 upregulates PROCR molecule in hPSC-derived HPs, but demonstrates minimal effect on *AHR* and *AHRR* expression. Although in cord blood expansion cultures, UM171 mostly decreases apoptosis without affecting proliferation of the most primitive HSCs, in hPSC-HP cultures, UM171 increased the number of cycling CD34^+^CD45^+^ cells, in addition to decreasing apoptosis.

Previous studies demonstrated that addition of UM171 or SR1 to hPSC differentiation cultures after hemogenic endothelium formation increases the number of CD34^+^CD45^+^ progenitors and CFCs^8,22^. In addition, inhibition of AHR with SR1 promotes NK cell formation and megakaryocytic lineage development from hPSCs^22,23^. In the present study, we revealed that despite some commonalities, SR1 and UM171 differently affected hematopoiesis from hPSCs. Although both small molecules increased NK cell production from hPSCs, UM171 mostly promoted expansion of CD34^+^CD45^+^CD7^+^ lymphoid progenitors, while SR1 accelerated differentiation of already formed NK cell progenitors^22^. UM171 was much more potent than SR1 in expansion of NK cells, allowing us to achieve up to 10-fold increase in NK cell output, over a 14-day period, as compared to an up to 2-fold increase that was reported in prior studies with SR1, over a 28-day period^22^. In contrast to SR1, UM171 predominantly enhanced production of G-CFCs with a CD34^+^CD41a^lo^CD45^+^ phenotype typical of early embryonic blood progenitors.

Overall, our studies revealed UM171 to be a potent small molecule for expansion of granulocytic and NK lymphoid progenitors from hPSCs. These findings will help to design protocols for scalable manufacturing of blood cells from hPSCs for transfusion and immunotherapy.

## Methods

### Human pluripotent stem cell and mouse OP9-DLL4 culture

The human ESC lines H1 (WA01) and WA09 (H9), the DF19-9-7T human fibroblast iPSC line, and the IISH2i-BM9 bone marrow-derived iPSC line were obtained from WICell Research Institute (Madison, WI), and maintained on vitronectin in E8 medium (WiCell). After reaching 75–80% confluence (about 3–4 days), cells were passaged by disassociation with 0.5 mM EDTA (Sigma) in PBS. E8 media was changed daily. OP9-DLL4 mouse feeder cells were maintained on 0.1% gelatin coated 10 cm dishes in OP9 growth media, consisting of α-MEM (Gibco), supplemented with 20% FBS (Hyclone). Cells were passaged at 75–80% confluency.

### Hematopoietic differentiation

Hematopoietic differentiation of hPSCs was performed using the two-dimensional hematopoietic differentiation protocol described previously^10^. In brief, hPSC colonies were singularized with 1X Tryple (Life Technologies) for 5 min. Singularized cells were then plated onto six-well plates coated with 0.5 μg/cm^2^ Coll IV (Sigma Aldrich) in E8 medium, supplemented with 10 μM Rho kinase inhibitor (Tocris Y-27632). Cells were plated at a density of 10,000 to 15,000 cells/cm^2^. After culture for 24 hours (day 0), E8 medium was changed to IF9S, supplemented with 50 ng/mL fibroblast growth factor (FGF2) (PeproTech), 50 ng/mL bone morphogenetic protein 4 (BMP4) (PeproTech), 15 ng/mL Activin A (PeproTech), and 2 mM lithium chloride (LiCl) (Sigma) for mesodermal development. TGF-β inhibitor (SB-431542, Cayman Chemicals) at 10μM concentration was added on day 2 of differentiation. Fresh cytokine cocktails in IF9S were prepared every two days, to induce stepwise differentiation through hematovascular precursors and hemogenic endothelium, into CD43 hematopoietic progenitors (day 2: 50 ng/ml FGF2 and 50 ng/ml VEGF; day 4 and 6: 50 ng/ml FGF2, VEGF, TPO, SCF, IL-6, and 10 ng/ml IL-3). Media was replaced on days 2 and 4, and added on top for day 6.

Differentiation was conducted for 9 days, first in hypoxic conditions (days 0–6), and then in normoxic conditions (days 6–9). Floating CD43 hematopoietic progenitor cells were collected at day 9, strained with a 70-μm cell strainer, and then used for experiments.

### Expansion of hematopoietic progenitors

Hematopoietic progenitors from Day 9 differentiation cultures were expanded in StemSpan^TM^-SFEM (Stem Cell Technologies), supplemented with 35 nM UM171(Xcess Biosciences), or equivalent volume of DMSO, and 100 ng/ml TPO, 100 ng/ml SCF, 100 ng/ml FLT3L, 50 ng/ml IL-6, and 10 ng/ml IL-3. Expansion cultures were supplemented with fresh StemSpan^TM^-SFEM (Stem Cell Technologies) media with the same cytokine concentrations, every 3 days. Cells were plated at up to 150,000 cells/ml. At day 5 or indicated time points, cells were collected for flow cytometric (FCM) analysis and CFU assay.

### Flow cytometry

Flow Cytometry was conducted using a MACSQuant® Analyzer 10 (Miltenyi Biotec), and the following antibodies: CD43-FITC(1G10), CD43-PerCP(1G10), CD235a-PE(HIR2), CD45-BV421(H130), CD45-PerCP(Hl30), CD94-Fitc(HP-3D9), CD38-FITC (HIT2), CD16APC(3G8), CD7-PE(MT701), CD16-FITC (3GB), CD42b-PE(HIP1), CD107a-FITC (H4A3) perforin-FITC (δG9), CD34 FITC (581), CD66BV421 (G10F5), CD16 PE (3G8), and CD14 PerCP (M5E2) from BD Pharmingen; CD41a-APC(REA386), CD45-APC(REA747), CD11b APC(REA713), CD201-PE (REA337), and anti-Ki67 (REA183) from Miltenyi Biotech, and CD56-PerCP(5.1H11), CD56-APC(5.1.H11), and CD45RA-APC (H1100) from BioLegend. All expansions were performed using ultra-low attachment surface, tissue culture plates (CORNING).

### Fluorescent Activated Cell Sorting (FACS)

Sorting was performed on a FACS Aria II cell sorter (BD). Cells of interest were harvested from differentiation cultures, and resuspended in MACS buffer (5% FBS, Gibco; 0.5 mM EDTA in PBS). The following antibodies were used: CD41a-APC, CD235a-PE, CD43-PerCP, CD45-PerCP, CD34-FITC (BD Pharmingen), and CD56-APC (BioLegend). Dead cells were counterstained with Ghost Violet 540 cell viability dye (TONBO Biosciences). Sorting gates were set with appropriate Fluorescent Minus One (FMO) controls, and only live cells were collected.

### Methylcellulose colony forming assay

Hematopoietic colony forming potential was assessed by combining cells of interest with 1.5 ml serum containing H4436 Methocult (Stem Cell Technologies). Cell suspensions were then transferred to 35-mm dishes, and cultured for 12–14 days at 37 °C. Hematopoietic colonies were scored according to cellular morphology, and CFC numbers were normalized to the number of cells plated (CFCs/10^4^) cells.

### Terminal differentiation into mature neutrophils

Neutrophil differentiation of DMSO or UM171 expanded hematopoietic progenitor cells was performed in IMDM (Gibco) medium, supplemented with 20% FBS (HyClone), on ultra-low attachment tissue culture plates (CORNING). Recombinant G-CSF (AMGEN) was added at a concentration of 100 ng/mL, for the first 3–4 days. Media was then topped up with an equivalent volume of fresh media, with 150–200 ng/mL G-CSF. After 7–9 days, floating cells were harvested, strained through a 70-μm cell strainers, and used for flow cytometry, cytospin, and functional assays.

### Phagocytosis assay by flow cytometry

To analyze the phagocytic capacity of neutrophils derived from DMSO and UM171 expanded hPSC derived HPs, we incubated these neutrophils with opsonized zymosan A fluorescein particles for 1 hour, at 37 °C or 4 °C (control). Samples were subsequently collected on ice, incubated with CD16-PE, CD66b-BV421(BD Pharmingen), and CD11b-APC (Miltenyi Biotech), and counterstained with Ghost violet 540 cell viability dye (TONBO Biosciences). Cells were then analyzed by flow cytometry.

### NK cell differentiation

NK differentiation of hematopoietic progenitor cells was performed in α-MEM (Gibco), supplemented with 20% FBS (HyClone), on mouse OP9-DLL4 expressing feeder layer, in two stages. In stage 1, hematopoietic progenitor cells were cultured for 7 days on OP9-DLL4, with 100 ng/ml FLT3L, 40 ng/ml SCF, and 5 ng/ml IL-7 (Peprotech), and 35 nM UM171 or equivalent volume DMSO. Cells were then transferred to fresh α-MEM media, supplemented with 10 ng/ml Il-2 and 5 ng/ml IL-15 (Peprotech), and with or without UM171 treatment. Media was topped up with one volume fresh media, every 3–4 days, and cultures were transferred to new OP9-DLL4 cells, every 7 days. After 14 to 21 days, cells were harvested for flow cytometry and other analyses. OP9-DLL4 were used at 75–80% confluency.

### *In vitro* cytotoxicity assay

DMSO and UM171 differentiated hPSC-derived NK cells were purified from NK differentiation cultures by FAC-sorting with CD56-APC (BioLegend). K562 tumor cells maintained in RPMI (Gibco), supplemented with 10% FBS were stained with PK467-GFP cell membrane marker (Sigma Aldrich), according to the manufacturer’s instructions. Purified CD56^+^ hPSC derived NK cells were then incubated with target K562 for 4 hours at 37 °C, at effector: target (E:T) ratios of 0.5:1,1:1, 3:1, 5:1, 10:1, and 20:1, in a final volume of 200 μl, in a 96 well plate. Following incubation, cells were collected, washed twice with MACS buffer, and then incubated with propidium iodide and annexin V-PE (BD, Biosciences), for flow based cytometric assay. Apoptosis/necrosis gates were set with appropriate Fluorescent Minus One (FMO) controls, and hPSC derived NK cells not cocultured with K562 were utilized for background subtraction.

### Interferon gamma assays

To assess interferon gamma production, FAC-sorted NK cells were stimulated for 5 hours with PMA and ionomyocin (1:500) Cell Activation Cocktail (BioLegend), or alternately K562 tumor target cells at a 2:1 ratio. Breferidin A (1:1000; Thermo Fisher) was added at the beginning of the stimulation. NK cells that were not stimulated served as experimental controls. At the completion of incubation, all cells were washed with MACS buffer (5% FBS, Gibco; 0.5 mM EDTA in PBS), and incubated with Ghost violet 540 cell viability dye (TONBO Biosciences) and CD56-PerCP (BioLegend), for 30 minutes. Subsequently, cells were permeabilized and stained with IFNγ antibody.

### Intracellular staining for detection of perforin

For assessment of intracellular perforin production, NK cells were incubated with Ghost violet 540 cell viability dye (TONBO Biosciences) and CD56-PerCP (BioLegend). After washing with MACS buffer, cells were then fixed and permeabilized, following instructions in the Intracellular Fixation and Permeabilization Buffer Set (Thermo Fisher). Cells were then incubated overnight with Perforin-FITC (BD Pharmingen), and analyzed by flow cytometry.

### Apoptosis and Proliferation Assays

For apoptosis detection, cells were stained with CD34-FITC, CD45-BV421, and CD43-APC (BD Pharmingen), along with PI and Annexin V-PE (BD Pharmingen), and analyzed by flow cytometry. To assess proliferation of hematopoietic progenitors, using cell proliferation antigen Ki-67, cells were incubated with CD34-PE, CD43-BV421 (BD Pharmingen), and Ghost violet 540 cell viability dye (TONBO Biosciences). Cells were then fixed and permeabilized with 70% ethanol, and stained with Ki67-APC (Miltenyi Biotech), according to the manufacturer protocol, and analyzed by flow cytometry. Cell cycle analysis of total hematopoietic progenitors was carried out using the FITC BrdU flow kit (BD Pharmingen), following manufacturer recommendations. On day four of HP expansion in HSC or lymphoid expansion conditions and on day 3 of NK differentiation, cells were labelled with BrdU. After 24 hours, samples were collected and incubated with CD34-PE, CD43-BV421(BD Pharmingen), and CD45-APC (Miltenyi Biotech); CD34-PE or 34 PerCP, CD45-BV421(BD Pharmingen), and CD41a-APC (Miltenyi Biotech); CD45-BV421, CD7-PE, and CD34-APC (BD Pharmingen); or CD45-BV421 and CD56-APC (BD Pharmingen), and then counterstained with Ghost violet 540 cell viability dye (TONBO Biosciences). This was followed by fixation, permeabilization, and a 1 hour incubation with DNase at 37 °C, to expose incorporated BrdU. After washing, cells were stained first with anti BrdU-FITC, followed by 7AAD, and flow cytometry was performed. CD34^+^CD43^+^CD45^+^ and CD34^+^CD43^+^CD45^+^CD41a^+^ hematopoietic progenitors, CD34^+^CD45^+^CD7^+^, lymphoid progenitors, or CD45^+^CD56^+^ NK cells were then analyzed for cell cycle kinetics and proliferation, through BrdU incorporation.

### RNA isolation and RNA-Seq data processing and analysis

Total RNA was isolated using RNeasy Micro Kit (Qiagen) and subjected to DNase digestion, according to the manufacturer’s protocol. Isolated RNAs were used for either quantitative RT-PCR or RNA sequencing. RNA purity and integrity was evaluated by capillary electrophoresis on the Bioanalyzer 2100 (Agilent Technologies, Santa Clara, CA). Samples were then prepared for sequencing using the Ligation Mediated Sequencing (LM-Seq) protocol, according to the published guidelines^24^. Final sample libraries were quantitated with the Life Technologies Qubit fluorometer, and sequenced on the Illumina HiSeq 3000 (SY-401-3001). Base-calling and demultiplexing were completed with the Illumina bcl2fastq2 utility, v2.17.1.14. Following quality assessment and filtering for adapter molecules and other sequencing artifacts, the sequencing reads were aligned to transcript sequences corresponding to hg19 human genome annotation. Bowtie v 1.1.2 was used, allowing two mismatches in a 25 bp seed and excluding reads with more than 200 alignments^25^. RSEM v 1.3.0 was used to estimate isoform or gene relative expression levels in units of “transcripts per million” (tpm), as well as posterior mean estimate of the “expected counts” (the non-normalized absolute number of reads assigned by RSEM to each isoform/gene)^26,27^. R statistical environment (R core team, 2014) was used at all stages of downstream data analysis. Count matrices were normalized using median normalization routine from EBSeq package^28^. EBSeq with 10 iterations and pooled variance estimate was used to call for differential expression. Genes that passed both Posterior Probability of Differential Expression >0.95 and fold change >2 were selected for further analysis. The accession number for the RNA-seq data reported in this paper is GEO: GSE128295 (https://www.ncbi.nlm.nih.gov/geo/query/acc.cgi?acc=GSE128295).

### Statistical Analysis

Statistical analysis was performed in Prism (GraphPad, San Diego, CA) using student t test. Data was reported as the mean ± SEM of at least 3 independent experiments. Results producing p < 0.05 were considered statistically significant.

## Supplementary information

Supplementary Figures
