# Supplementary material for: UM171 expands distinct types of myeloid and NK progenitors from human pluripotent stem cells
Source: Sci Rep. 2019 Apr 29;9:6622. doi: 10.1038/s41598-019-43054-4 (PMC6488662; doi:10.1038/s41598-019-43054-4)
Supplement: Supplementary file 1 — Supplementary Figures [file 41598_2019_43054_MOESM1_ESM.pdf]

Supplemental Materials for

Walatta-Tseyon Mesquitta, Matthew Wandsnider, HyunJun Kang, James Thomson, Oleg Moskvin, Kran Suknuntha, and Igor I. Slukvin

UM171 expands distinct types of myeloid and NK progenitors from human pluripotent stem cells

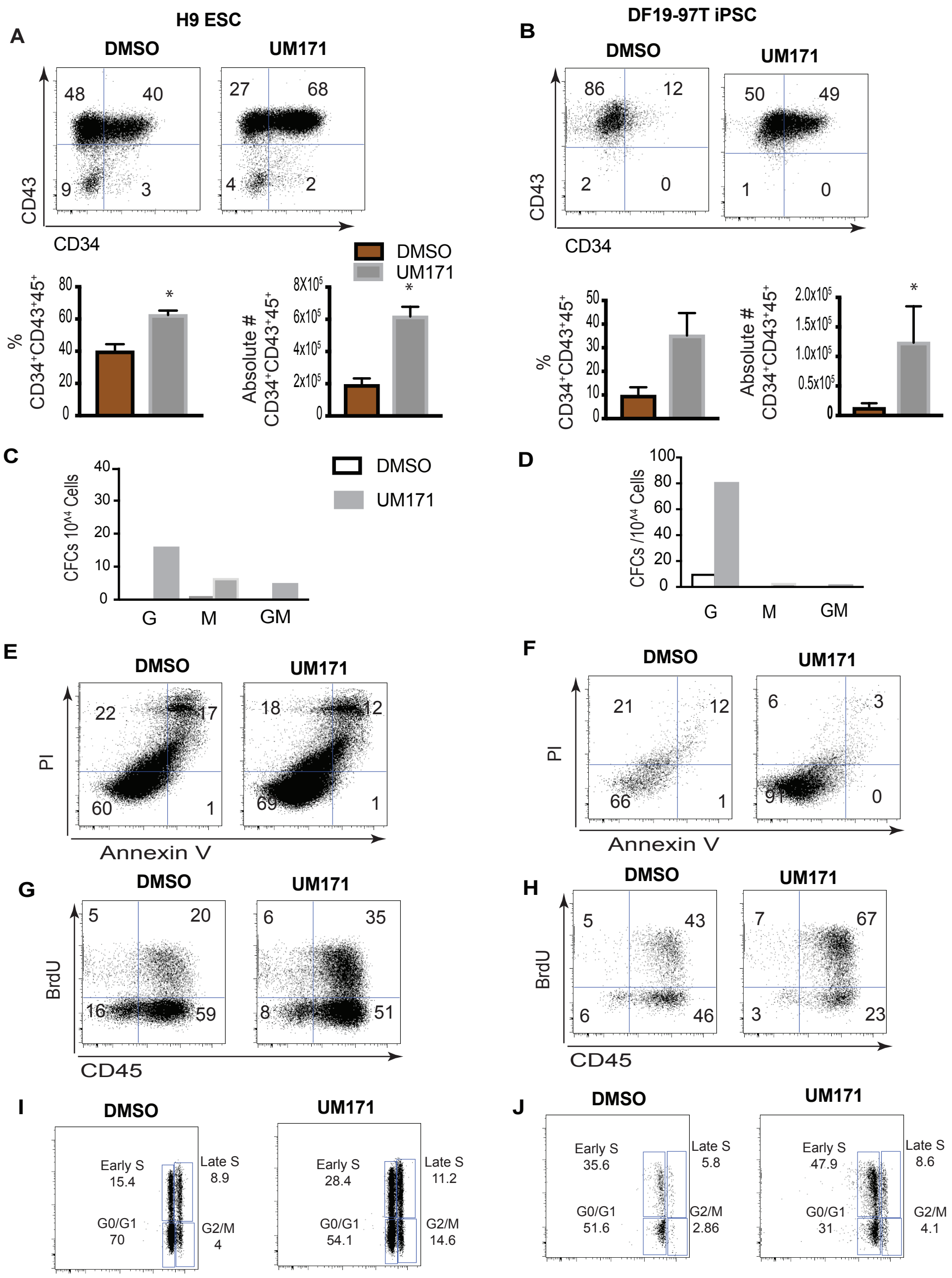

Figure S1. UM171 effect on expansion of CD34⁺CD43⁺ HPs derived from H9 hESCs and DF19-9-7T fibroblast-derived iPSCs. (A)-(B) Representative dot plots showing CD34 and CD43 expression following 5 days of expansion hPSC-derived HPs with UM171 or DMSO (control). Bar graphs showing UM171 effect on % and absolute numbers of $CD34^+CD43^+CD45^+$ HPs. Results are mean$\pm$SEM for 3 independent experiments. *$p<0.05$. (C)-(D) Graphs showing CFC potential of expanded cells. (E)-(F) Representative dot plots showing apoptosis evaluation using annexin V staining, in cultures of hPSC-derived HPs expanded for 5 days. Dot plots show $CD34^+CD43^+$ gated cells. (G)-(H) Representative dot plots showing assessment of proliferative potential of $CD34^+CD45^+$ HPs expanded for 5 days with UM171 or DMSO, using (G-H) BrdU assay and (I,J) cell cycle analysis. (A), (C), (E), (G), (I) showing H9 hESCs and (B), (D), (F), (H), (J) showing DF19-9-7T iPSCs.

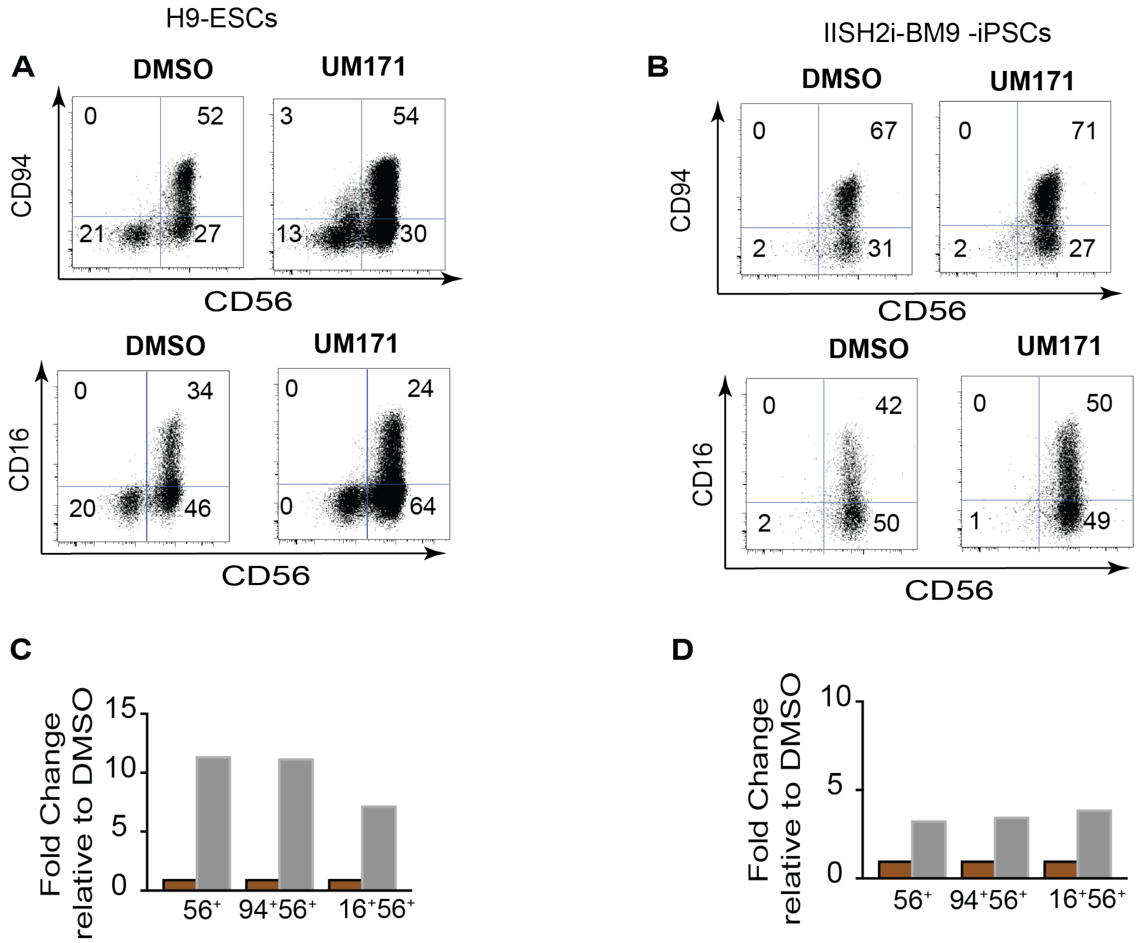

Figure S2. Effect of UM171 on NK cell production from CD34^{+}CD43^{+} HPs derived from H9 hESC and IISH2i-BM9 derived from bone marrow iPSCs. (A)-(B) Representative flow cytometry dot plots displaying NK cell differentiation. (C)-(D) Bar graphs showing fold changes in absolute numbers of NK cell subsets. Representative experiment is shown.
